# *Letter:* Restricted Access to Electroconvulsive Therapy in Pediatric and Adolescent Patients—Clinical and Familial Consequences: Case Report

**DOI:** 10.1177/10445463261478543

**Published:** 2026-08-08

**Authors:** Jarod Lowe, Joshua Ryan Smith

**Affiliations:** 1 Vanderbilt University School of Medicine, Nashville, Tennessee, USA.; 2 Vanderbilt Kennedy Center, Vanderbilt University, Nashville, Tennessee, USA.; 3 Division of Child and Adolescent Psychiatry, Department of Psychiatry and Behavioral Sciences, Vanderbilt University Medical Center at Village of Vanderbilt, Nashville, Tennessee, USA.

Electroconvulsive therapy (ECT) is a procedure that uses electrical current to induce a brief generalized seizure to treat severe neuropsychiatric illnesses.^1^ ECT is well tolerated and elicits high response rates for catatonia, treatment-resistant mood, and psychotic disorders.^1^ Although pediatric data are limited, available studies report favorable outcomes,^2^ including in patients with autism spectrum disorder (ASD) and developmental regression—conditions that have lower response rates to conventional treatments.^3^ Despite this and for various reasons, there is limited access to ECT for pediatric and adolescent patients. In the case presented, such barriers delayed care, resulted in significant morbidity for the child, and substantially disrupted the family’s life as they sought effective treatment for their daughter.

JB is a 13-year-old girl with CHD8-related neurodevelopmental disorder (CHD8-NDD), a rare autosomal dominant condition associated with developmental delay, intellectual disability, ASD, neuropsychiatric issues, sleep disturbance, and gastrointestinal issues.^4^ Despite her diagnosis, she integrated well with peers, participating in ballet and swimming, and speaking both English and Spanish. In 2023, she experienced sudden regression, becoming mute, staring unresponsively for prolonged periods, attempting to elope, and losing the ability to perform activities of daily living. Just before age 11, she started to display limb posturing and perseverative behaviors like object shaking, which were attributed initially to her neurodevelopmental condition.

Catatonia was considered as a diagnosis, but an initial lorazepam challenge performed outside an active episode was interpreted as negative, and the diagnosis was questioned. At the time, JB and her family resided in Texas, where ECT is prohibited for individuals younger than 16 years.^5^ Her condition continued to deteriorate, with escalating dangerous aggression, worsening oral intake leading to significant nutritional compromise and withdrawal from school at age 12. Escalating medical decline combined with newly imposed workplace restrictions led to the father ending his 18-year career in aerospace engineering to care for his daughter. The family left their home to seek treatment, relocating through temporary housing with no income—a situation that persists to the date of this report.

The family pursued outside consultation with Vanderbilt University Medical Center, where catatonia was reconsidered, and ECT was recommended for JB. Unlike Texas’s aged-based exclusion of ECT for those under 16, Tennessee’s laws on its use in that population focus more on clinical justification and consent.^6^ Consistent with Tennessee state law, JB underwent evaluation by the treating team, plus an independent court-appointed child and adolescent psychiatric evaluation as required by Tenn. Code Ann. § 33-8-308, multidisciplinary case review, and judicial authorization in juvenile court per § 33-8-305. She then received 11 ECT treatments over 40 days, with her Catatonia Rating Scale improving from a score of 20 to 2, indicating significant improvement in catatonic symptoms (see chronological progress in Table 1). While she has not returned to her premorbid baseline due to the progression of her condition, she has since demonstrated behavioral improvement and returned to school.

JB’s case presents how age-based statutory exclusions, such as those in Texas, affect the quality of life for pediatric patients with catatonia and their families through policy reform. Despite literature supporting the safety and efficacy of ECT for pediatric neuropsychiatric disease, persistent misconceptions and stigma continue to have undue influence on restrictive legislation, access, and provider comfort with ECT.^2,7^ To prevent cases like JB’s, reform of state policies is warranted to allow timely supervised access to ECT for children and adolescents with medication-resistant psychiatric disease.

## Figures and Tables

**Table 1.** Timeline of JB’s Progression and Actions Taken by Her Family

Date	Medication	ECT number	BFCRS KCS KCP	Clinical course	Family action
September 2023	—	—	—	Early symptoms of mutism, anorexia, perseverations begin	Family first takes JB to the doctor for symptoms.
February 2024	Cyproheptadine (for cyclic vomiting)			Mutism and dysphagia progress, serial EEG’s conducted	Catatonia was first considered as a diagnosis
August 2024	Olanzapine, quetiapine, guanfacine, hydroxyzine,			JB undergoes inpatient testing	Father permanently working from home.
December 2024	—			—	Mother quits job in order to take care of JB
February 2025				JB first hospitalized for self-injurious behavior; Agitation and aggression worsen	Agitation and self-injury attributed to antipsychotics and they are discontinued; JB removed from school due to illness; father quits job to take care of JB
March 2025	Aripiprazole			Hospitalized 2^nd^ time for self-injurious behavior, aggression, anorexia	Family has to hire sitter to help take care of nanny
April 2025	Gabapentin, Aripiprazole			Hospitalized 3^rd^ and 4^th^ time	Family calls 911 multiple times for aggressive behavior
May 2025	Cyproheptadine (for appetite stimulation)			Hospitalized 5^th^ and 6^th^ time	
June 2025	Lorazepam challenge administered with minimal response			Hospitalized 7^th^ time	
July 2025	50 mg Chlorpromazine BID		BFCRS: 21	—	JB presents to Vanderbilt clinic for evaluation of catatonia
			KCS: 46		
			KCP: 4		
August 2025	2 mg Lorazepam TID	1	BFCRS: 20		Family living in charity housing, but rejected due to behavior issues
	2 mg Lorazepam PRN		KCS: 50		
	12.5 mg Clozapine BID		KCP: 2		
	50 mg Chlorpromazine BID	2	BFCRS: 16	JB could dress and dry herself after a shower, but scratched and bit mother without provocation	Family living from homestay to hotel week by week
	750 mg/1000 mg		KCS: 42		
	Valproate BID		KCP: 1		
		3	BFCRS: 14	More bearable, more consciously present, less aggression and self-harm	
			KCS: 40		—
			KCP: 1		
		4	BFCRS: 14	—	
			KCS: 40		
			KCP: 1		
		5	BFCRS: 10	Parents report positive trajectory, less intense aggression	
			KCS: 26		
			KCP: 1		
	2 mg Lorazepam PRN	6, 7	BFCRS: 10	Food obsession began, with aggressive behavior	
	300 mg Lithium BID		KCS: 26		
	50 mg Chlorpromazine BID		KCP: 1		
	750 mg/1000 mg	8	BFCRS: 8	Father reports she is cheerful, singing and dancing and conversing	
	Valproate BID		KCS: 22		
			KCP: 1		
September 2025	1.5 mg Lorazepam TID	9	BFCRS: 4	Sleep issues improved, occasional agitation episodes	—
	+0.5 mg before bed		KCS: 18		
	50 mg Chlorpromazine BID		KCP: 0		
	750 mg/1000 mg				
	Valproate BID				
September 2025–February 2026	3 mg Lorazepam TID	10^a^	BFCRS: 2	Stable behavior with occasional aggression and low cognitive level; restarted school in January	Family still unemployed with no income, can’t move back home to Texas because ECT is not available
	50 mg Chlorpromazine BID		KCS: 4		
	750 mg/1000 mg		KCP: 0		
	Valproate BID				

a Maintenance ECT is continually done biweekly to maintain treatment of catatonia.

BFCRS, Bush-Francis Catatonia Rating Scale; KCS, Kanner Catatonia Scale; KCP, Kanner Catatonia Physical Examination.
